# Silicon Nitride and Hydrogenated Silicon Nitride Thin Films: A Review of Fabrication Methods and Applications

**DOI:** 10.3390/ma14195658

**Published:** 2021-09-28

**Authors:** Nikolett Hegedüs, Katalin Balázsi, Csaba Balázsi

**Affiliations:** 1 Centre for Energy Research, Institute for Technical Physics and Materials Science, 1121 Budapest, Hungary; balazsi.katalin@ek-cer.hu (K.B.); balazsi.csaba@ek-cer.hu (C.B.); 2 Doctoral School of Materials Science and Technologies, Óbuda University, 1030 Budapest, Hungary; 3 Guardian Orosháza Ltd., 5900 Orosháza, Hungary

**Keywords:** SiNx thin films, chemical vapor deposition, radio frequency sputtering, atomic layer deposition

## Abstract

Silicon nitride (SiNx) and hydrogenated silicon nitride (SiNx:H) thin films enjoy widespread scientific interest across multiple application fields. Exceptional combination of optical, mechanical, and thermal properties allows for their utilization in several industries, from solar and semiconductor to coated glass production. The wide bandgap (~5.2 eV) of thin films allows for its optoelectronic application, while the SiNx layers could act as passivation antireflective layers or as a host matrix for silicon nano-inclusions (Si-ni) for solar cell devices. In addition, high water-impermeability of SiNx makes it a potential candidate for barrier layers of organic light emission diodes (OLEDs). This work presents a review of the state-of-the-art process techniques and applications of SiNx and SiNx:H thin films. We focus on the trends and latest achievements of various deposition processes of recent years. Historically, different kinds of chemical vapor deposition (CVD), such as plasma enhanced (PE-CVD) or hot wire (HW-CVD), as well as electron cyclotron resonance (ECR), are the most common deposition methods, while physical vapor deposition (PVD), which is primarily sputtering, is also widely used. Besides these fabrication methods, atomic layer deposition (ALD) is an emerging technology due to the fact that it is able to control the deposition at the atomic level and provide extremely thin SiNx layers. The application of these three deposition methods is compared, while special attention is paid to the effect of the fabrication method on the properties of SiNx thin films, particularly the optical, mechanical, and thermal properties.

## 1. Introduction

Silicon nitride and hydrogenated silicon nitride thin films attract widespread scientific interest across multiple application fields. They are characterized by an outstanding combination of optical, mechanical, and thermal properties, allowing for their application in different industrial branches. In solar cell applications, they serve as antireflection and passivating coatings [1,2]. The standard method for opening SiNx passivating films is screen printing [3]. However, Bailly et al. [4] reported laser ablation as a promising alternative method of opening SiNx layers on alkaline-textured crystalline Si to make contact with Si solar cells. This indirect method allows for the mitigation of surface defects, thus enhancing the performance of the device. Since solar cells typically consist of multilayer structures, including the substrate, the layer responsible for sealing, and the transparent conductive oxide layer, light management involves the reduction of reflectivity loss not only at the glass surface but also at the other interfaces. Due to the tunable refractive index of SiNx, it can effectively mitigate the reflectivity loss at the glass/TCO surface on scalable industrial sizes that extend up to the theoretical limit of 1.6% [5]. Moreover, Si-rich SiNx layers could act as host materials for nanometer-sized Si crystals (NCs). Multilayer stacks, including alternating silicon-rich nitride (SRN) layers with embedded Si NCs and thin Si_3_N_4_ layers, are advantageous for photovoltaic applications since the thin Si_3_N_4_ layers enable better electrical conductivity and simultaneously allow for the growth of Si NCs with the desired size [6].

In the heterostructure field effect transistor (HFET) application field, in which GaN is utilized, a crucial role is played by the heterostructure surface passivation in order to decrease the influence of dispersion [7]. Capacitance-voltage [C(V)] characterization of the boundary of the heterostructure and the passivation thin film revealed that SiNx layers deposited by different methods are able to reduce the density of interface traps; however, the preparation method of the layer affects its passivation properties [8].

Photonic integrated circuits (PIC) could have a significant contribution to meet the ever-growing requirements of communication networks. Despite the fact that the high price of PICs compared to that of electronic-integrated circuits currently acts as a barrier for its application, scientific interest in this technology is continuously emerging [9]. Sharma et al. [10] provided an overview of the latest achievements of SiNx-based PICs, highlighting the benefits of these devices such as the small signal loss or the ability to work with wide-wavelength ranges. Frigg et al. [11] found that SiNx thin films deposited by direct current sputtering is able to further decrease the loss, which is attributed to the lack of hydrogen bonds compared to its other chemical vapor deposited counterparts.

Improvement of non-volatile memories is particularly driven by the growing popularity of mobile electronic devices. In this field, SiNx is applied as a dielectric layer to trap charges. To avoid heat induced charge migration from one trap to another, as well as to avoid the shift of the threshold voltage due to non-eliminated electrons and holes, Mine et al. [12] suggested the application of silicon-rich silicon nitride thin films in non-volatile memory devices.

High hardness as well as the attractiveness of other mechanical and tribological properties make SiNx thin films potential candidates for hard coating applications under challenging environmental and thermal conditions [13]. Nano-indentation, micro-scratching, and tribological tests proved the hardness of SiNx to be similar to that of sintered Si_3_N_4_ and elastic modulus near to that of cobalt chromium. Moreover the wear resistance of SiNx even exceeded that of cobalt chromium, approaching the wear resistance of bulk silicon nitride [14].

Apart from the photovoltaic devices, which have enhanced transmittance in the visible wavelength range and increased reflectance in the far infrared range, SiNx layers are a suitable material for low-emissivity (LowE) glass coatings, which are utilized in architectural glazings to mitigate heat losses in architectural applications. In this case, multilayer stacks, which usually composed of dielectric/metal/dielectric layers, are deposited on the glass surface, where the role of the silver layer is to reflect IR radiation back to the inside of the building and the dielectric materials protect the silver as well as act as antireflection layers. SiNx thin films usually deposited by magnetron sputtering are often used as dielectric layers in such applications [15].

Stochiometric silicon nitride (Si_3_N_4_) exists in three different crystallographic structures, namely α, β, and γ phases [16]. While the most common α and β phases can be synthesized under normal pressure, the formation of the γ phase requires high pressure and temperature conditions [17]. Amorphous silicon nitride (a-SiNx) presents a wide range of layer properties that are a function of the structure and bonding configuration as well as of the composition of the film. Tailoring the refractive index of a-SiNx is feasible by controlling the N/Si ratio of the films [18,19,20].

Fabrication technologies of SiNx layers are generally divided into two main techniques, namely the chemical vapor deposition (CVD) technique and the physical vapor deposition (PVD) technique, which are based on the types of involved reactions during deposition. In the case of CVD methods, the material introduced to the deposition chamber in the gas phase is deposited as a result of chemical reactions on the substrate surface where a thin film is grown. Additionally, a common feature of PVD methods is that the material, which is initially typically in the solid phase, is transformed to the gas phase, after which the material then returns to a solid phase by creating a layer on the desired substrate. In the case of PVD techniques, sputtering is the method predominantly used, while for CVD technologies, several different processes, e.g., hot wire (HW-CVD) [21,22], expanded thermal plasma (ETP-CVD) [23], electron cyclotron resonance (ECR-CVD) [24,25,26,27], and both plasma enhanced (PE-CVD) [28,29,30] and remote plasma enhanced (RPE-CVD) [31], are applied for the deposition of silicon nitride thin films. Due to the demand for extraordinary thin SiNx layers with precisely controlled composition and layer properties, increasing scientific interest appeared for a subset of CVD, namely the atomic layer chemical vapor deposition (ALCVD) or atomic layer deposition (ALD). In these processes, the thin film is formed on the substrate by atomic layers using chemical reactions in the gas atmosphere. For the deposition of SiNx thin films that are thermal [32,33,34], plasma-assisted (PA), and plasma-enhanced (PE) [35,36,37,38], ALD are the method most often used. Considering the growing scientific interest ALD methods are receiving in recent years, we discuss them separately from other CVD methods.

Kaloyeros et al. [39,40] provided excellent review articles on the field of SiNx and SiNx-rich thin films, including SiNx with carbon inclusions as well as hydrogenated SiNx thin films. They paid special attention to the precursor gas chemistry by overviewing the properties (e.g., bond dissociation energies) of the most common as well as most rarely used Si and N sources. They have emphasized that recently a new trend appeared in processing technology that aims to lower deposition temperature. Guided by this objective, Si–N bonds and C-containing precursors were recognized and begun to be applied in recent years. In addition, they highlighted that precursors which are able to react with substrate defects are gaining more scientific interest.

The aim of this study, on one hand, is to provide an overview on the latest achievements and trends of various deposition techniques of silicon nitride and hydrogenated silicon nitride. On the other hand, our intent is also to capture the effect of the preparation technique on the film properties, particularly the optical properties. The latter mentioned correlation between process parameters and film properties is considered to be useful as a detailed analysis of the composition and microstructure of SiN_x_ and SiN_x_:H thin films is often not available in the literature.

## 2. Chemical Vapor Deposition

**Figure 1:**
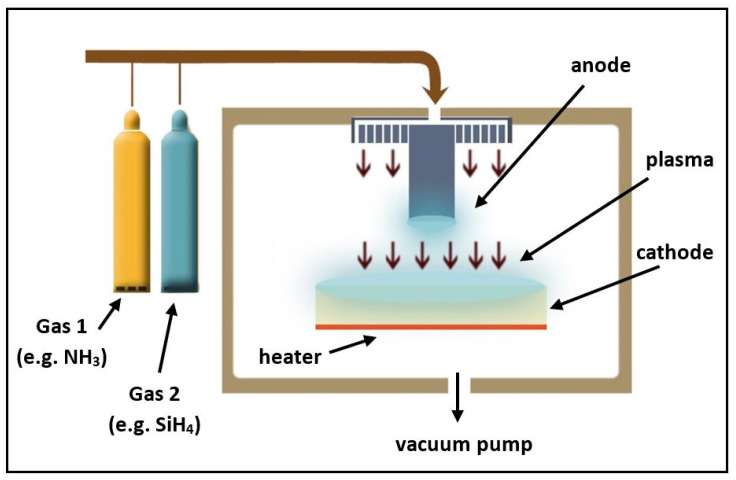
Schematic drawing of a PE-CVD reactor.

CVD is a widespread vacuum deposition method to prepare high quality thin films, where the desired film is created by the chemical reactions between precursor gases (raw materials) on the substrate surface. Many variants of CVD technology are known, of which the hot wire (HW-CVD) [21], expanded thermal plasma (ETP-CVD) [23], electron cyclotron resonance (ECR-CVD) [24], and plasma-enhanced (PE-CVD) [28] types are the most common for the deposition of SiNx and SiNx:H thin films. Figure 1 presents a schematic overview of a typical PE-CVD reactor.

### 2.1. Precursor Gas Atmosphere and Deposition Temperature

Regardless of which of the abovementioned CVD methods are applied, one of the key governing factors of film properties is the precursor gas atmosphere. Torchynska et al. [41] investigated the photoluminescence and structural characteristics of Si-rich silicon nitride layers deposited by PE-CVD. They applied SiH_4_ and NH_3_ as precursor gases, with the R = [ammonia]/[silane] flow ratio varying between 0.45 and 1.0. Their systematic study on the layer properties revealed that the pattern of photoluminescence (PL) spectra is governed by the precursor gas ratio. They found that the PL peak is shifted down from 2.7–3.0 eV to 1.9 eV, while R is decreased from 1.0 to 0.63.

Lee et al. [42] performed a comparative study on the passivation and optical characteristics of SiNx:H layers fabricated by PE-CVD from three different precursor mixtures: SiH_4_ + NH_3_ + N_2_ and SiH_4_ + NH_3_, SiH_4_ + N_2_. In terms of optical (antireflection) properties, they found minor changes between the reflectance spectra at the short wavelength range (300–550 nm). The absorption coefficient showed significant variations in the case of the different gas mixtures. The SiH_4_ + NH_3_ + N_2_ atmosphere was proved to result in the lowest absorption coefficient, while the highest value was obtained for SiH_4_ + N_2_. In the case of the passivation properties, they studied the lifetime of the minority carrier and the capacitance–voltage (C–V) correlation as a function of gas mixtures. In conclusion, films deposited from the SiH_4_ + NH_3_ + N_2_ gas mixture were proved to be the best choice according to the optical and passivation properties for solar cells application.

Jasruddin et al. [43] systematically investigated the effect of ammonia concentration with two specified silane gas flows on the optical bandgap and dark conductivity of PECVD SiNx:H layers, applying a gas mixture of ammonia, hydrogen, and silane gases. The highest optical band gap and lowest dark conductivity they have achieved were 3.97 eV and $1.05\times 10^{-12}\;\frac{S}{{\mathrm{cm}}^{2}}$, respectively. These parameters were obtained by the lower (5 sccm) silane gas flow and with 25% ammonia gas fraction. A similar trend was found by Vet et al. [44] who deposited SiNx:H layers by the same method, varying the gas ratio of silane while the amount of ammonia gas was fixed. In accordance with the results of Jasruddin et al., they have found that while the silane gas flow was decreased, the optical band gap was increased.

Besides the precursor gas atmosphere, the deposition temperature was proved to be an influencing factor of several layer properties. K. Jhansirani et al. [45] studied the optical behavior and chemical bonds of silicon nitride layers deposited at temperatures 750, 800, and 850 °C. They found a rising trend of the refractive index while the deposition temperature was increased, which could be explained by the densified growth of the layer at increased temperatures. Furthermore they studied the evolution of the Fourier transformed infrared spectroscopy (FTIR) peak corresponding to the Si-N-Si stretching mode. The move of the peak location from 942 to 881 cm^−1^ as well as the increase of the full width at the half maxima of the peak, with respect to the increase of temperature, were revealed. Reflectance calculations proved that the deposition temperature of 800 °C is optimal for fabrication antireflection coating for solar cell applications. Conversely, different applications require much lower deposition temperatures of SiNx thin films. For instance, plastic substrates for organic electronic devices (such as organic light-emitting diode (OLED)) are receiving more attention, wherein one of the key challenges concern that plastics are permeable for gases found in the atmosphere, such as water vapor or oxygen. Alpuim et al. [46] investigated SiNx multilayer stacks fabricated by HW-CVD and the special treatment by means of Ar-plasma between single layers as potential permeation barrier layers for these applications. Approximately 30 eV ion energy and the low-temperature (~100 °C) deposition process were found to result in a minimal water vapor transmission rate (WVTR) for polyethylene terephthalate coated by SiNx multilayer stacks.

### 2.2. Mechanical Properties

Outstanding mechanical properties of SiNx thin films allow for its use in component fabrication of microelectromechanical systems (MEMS) [47], where they act as carrier membranes. In these devices, the intrinsic state as well as the magnitude of film stress were proved to directly affect the performance and reliability of the devices. Toivola et al. [48] studied the deposition stress and hardness of low-pressure CVD SiNx films by means of ammonia (NH_3_) and dichlorosilane (DCS) at different deposition temperatures (813–876 °C), pressures (208–615 mTorr), and [DCS]/[NH_3_] precursor gas ratios (4.5–8.7). Deposition (tensile) stress was varied in the range of 135–235 MPa and showed a decreasing trend with higher fabrication temperatures, pressures, and precursor ratios. Nanoindentation tests utilizing a Berkovich (three-sided diamond-shape pyramid) tip revealed that the hardness of the films (28.0 ± 2.3 GPa) is almost independent from the film stress obtained by the fabrication at room temperature.

Slightly lower hardness was found by Taylor [49] who investigated the Si/N ratio effect on the mechanical behavior of PECVD SiNx layers. Their work revealed that changing the ratio of the precursor flow (e.g., NH_3_, N_2_, and SiH_4_), while all other processing parameters were kept constant led to SiNx films with different stoichiometries with the Si/N ratio varying between 1.04 and 1.53. Measured hardness values were in the range of 16.1–19.8 GPa and showed an increasing trend against the increasing Si/N ratio.

King et al. [50] studied the hardness of SiNx thin films among other mechanical parameters. It was revealed that the distribution of H-bonds between Si–H and N–H bonds affects the hardness of the layer. According to their results, hardness varied between 13 and 23 GPa, while the densities of Si–H and N–H bonds were in the range of 0.5–1.2 × 10^22^ cm^−3^ and 0.8–2.0 × 10^22^ cm^−3^, respectively. Higher N–H bond density was found to contribute to enhanced hardness, while increasing Si–H bond densities resulted in decreased hardness values. For the investigation of apparent fracture toughness, they applied nanoindentation (K_ind_) and examined SiNx:H films with film stress varying from 300 MPa tensile to 950 MPa compressive stress. K_ind_ was found to vary in a wide range from $0.2\;\mathrm{to}\;8.0\;\mathrm{MPa}\;\cdot \sqrt{m}$, which was attributed to mainly the intrinsic stress of the films. Near linear correlation was revealed between K_ind_ and the intrinsic film stress. Using empirical values from the linear regression analysis, the $1.8\;\pm 0.7\;\mathrm{MPa}\;\cdot \sqrt{m}$ stress-free fracture toughness was also determined for SiNx:H films fabricated by PECVD.

Since SiNx layers are increasingly used as flexible membranes, characterization of the two important elastic moduli, namely the Poisson’s ratio (*v*) and Young’s modulus (E), is necessary. Direct measurements of these parameters for submicron thick layers are unreachable for most of the conventional techniques. Brillouin light scattering (BLS) serves as a non-destructive tool to overcome these difficulties [51]. BLS was exploited by Carlotti et al. [52] to study E and *v* for dielectric films such as SiNx, silicon oxynitride, and silicon oxide fabricated by various techniques on the (100) Si-substrate. Of all the layers they examined, E and *v* were proved to be the highest for LPCVD SiNx layers, with values of 256 GPa and 0.28, respectively.

### 2.3. Thermal Properties

The ability to dissipate heat is frequently a restrictive factor of microelectronic devices, therefore high thermal conductivity is desirable for thin films such as SiNx, as used in this application field. Mastrangelo et al. [53] investigated thermal properties of low-residual stress LPCVD SiNx layers by means of composite microbridge resistors built up from two layers. In this arrangement, the composite bridge consisted of SiNx as the bottom layer and polycrystalline silicon as the top layer, and each bridge laid on two 3 µm thick phosphosilicate glass (PSG) pedestals. They found a thermal conductivity of $3.2\pm 0.5\;{\mathrm{Wm}}^{-1}K^{-1}$, thermal diffusivity of $1.3\times 10^{-2}\pm 1\times 10^{-3}\;{\mathrm{cm}}^{2}s^{-1}$, and heat capacity of $0.7\pm 0.1\;{\mathrm{Jg}}^{-1}K^{-1}$. Table 1 shows the thermal conductivity values reported in the literature for other commonly used materials in microelectronic devices for comparison.

The effective transverse thermal conductivity of dielectric layers was proved to decrease significantly while the film thickness is reduced, which is attributed to the interfacial thermal resistance developed at the interface of the thin film and Si substrate [59]. The research of Griffin et al. [60] focuses on the effect of the CVD SiNx film thickness and the temperature on its experimentally determined thermal conductivity. It was revealed that the effective transverse thermal conductivity of SiNx layers decreases substantially against the reduced film thickness, while conductivity increases from $2.0\;\mathrm{to}\;2.5{\;Wm}^{-1}K^{-1}$ due to the increase of temperature from 70 to 200 °C.

A similar correlation between temperature and thermal conductivity was found by Lee et al. [61] who characterized 20–300 nm thick PECVD SiNx films using the 3ω method. In this process, a metal strip is in contact with the thin film (acting as heater and thermometer). The strip heats the sample periodically by means of AC current, which causes a delayed periodic temperature response of the sample. Then, the amplitude-modulated signal of the voltage drop across the strip is analyzed. The name of the method (3ω) refers to the fact that if the angular frequency of the current applied to the strip is signed with ω, then a small part at the third harmonic 3ω of the voltage drop signal is used to determine the thermal properties of the thin film. Lee et al. found increasing thermal conductivity from ~0.25 to ~0.7 ${\mathrm{Wm}}^{-1}K^{-1}$, while the temperature was increased from 77 to 350 K. They compared their results with data of atmospheric pressure CVD (APCVD) SiNx layers. It was revealed that thermal conductivity of even relatively thick PECVD SiNx is reduced relative to APCVD SiNx films with a temperature-independent factor of 2.

A significant effect of layer thickness on thermal conductivity was also proved by the study of Bogner et al. [62] who have investigated PECVD SiNx films with various thicknesses. Thermal conductivity of the layer characterized by the 3ω technique was found to vary from 0.8 to 1.7 ${\mathrm{Wm}}^{-1}K^{-1}$, while the film thickness changed from 298 to 1001 nm. The obtained value was significantly lower than that reported for bulk Si_3_N_4_ [63].

### 2.4. Optical Properties

One of the widest application fields of SiNx thin films concern solar cells. In these photovoltaic applications, SiNx layers act as antireflective coatings (ARC) by means of its tunable optical properties. Besides a single layer, stacking ones can be also employed by adjusting the thickness and refractive index of the layers. Joshi et al. [64] investigated the refractive index of LPCVD and PECVD SiNx layers by means of ellipsometry. In the case of the LPCVD technique, the refractive index of 2.01 was found to be characteristic, while for PECVD, the effect of the precursor flow ratio, thus the Si/N atomic concentration ratio of the films on the refractive index, was experienced. It was revealed that the decrease of the silane to ammonia ratio (R_x_) led to decreasing the Si/N atomic concentration from 0.92 to 0.70 and thus resulted in a decreasing trend of the refractive index as well from 1.96 to 1.8. A similar correlation was found by Lowe et al. [65] who investigated the optical properties of SiNx films prepared by the PECVD technique, varying the ammonia to silane flow ratio from 0 to 20. They found that the refractive index was reduced monotonously from ~2.7 to ~1.6, while the N/Si atomic concentration ratio was increased in the range of 0–1.2. The work of Maeda et al. [66] highlighted that in addition to the N/Si atomic concentration ratio, substrate temperature and radio frequency (rf) power density also play significant roles in determining the refractive index of PECVD-deposited SiNx layers. In terms of substrate temperature, the refractive index of the layers were increased from ~1.91 to ~1.98, while substrate temperature was increased from 250 °C to 350 °C. Their results showed that the refractive index of the thin films was able to be tuned over an even wider range by adjusting the rf power density. In applying the substrate temperature of 350 °C, an increase of the refractive index from 1.98 to 2.09 was experienced, while the rf power density was changed from 0.5 to 1.5 W/cm^2^. Of the samples they studied, those with a refractive index of 1.98, N/Si ratio of 1.32, and density of 2.8 g/cm^3^ were proved to be optimal for the fabrication of SiNx layers as final passivation films for silicon-integrated circuit technology. A similar trend of the refractive index was found by Mei et al. [67] who have investigated the optical behavior of as-deposited and annealed PECVD SiNx layers, varying the deposition temperature from 300 °C to 500 ^o^C. The refractive index of the layers at a certain wavelength (632.8 nm) showed an increasing trend from 2.05 to 2.11, while the deposition temperature was raised from 300 °C to 500 °C. Investigation of SiNx films annealed for 30 min at 690 °C revealed that the refractive index slightly decreases due to annealing, while the correlation between the deposition temperature and refractive index remains after annealing.

Charifi et al. [68] characterized how the ammonia to silane ratio (R = [NH_3_]/[SiH_4_] in the range of 0.5 to 5 influences the refractive index of SiNx layers prepared by ECR-PECVD. It was revealed that the refractive index at 633 nm increased from 1.95 to 3.35, while R was decreased from 5 to 0.5. This trend can be interpreted by the fact that increasing silicon content of the layers due to higher silane concentration of the precursor gas mixture (R < 2) results in the reflective index approaching ~3.42, which is a value characteristic for c-Si, while lower silane content (R > 2) leads to a quasi-constant refractive index close to that of Si_3_N_4_ (n = ~1.97).

An ideal SiNx layer, such as ARC of crystalline silicon solar cells, should enhance transmittance, while, in parallel, suppressed surface recombination is also desirable. The key challenge in this area is that the high limit of effective surface recombination velocity (S_eff,UL_) can be reduced at the cost of increasing the refractive index of the SiNx layers [69]. Since SiNx layers with a high refractive index absorb well light in the short wavelength range, thus reducing the optical transmission, the trade-off is outlined between the transmittance and surface passivation. The work of Wan et al. [70] aimed to optimize these two parameters simultaneously by circumventing the abovementioned trade-off. They investigated SiNx layers prepared by the PECVD method, varying the temperature and pressure of the fabrication, as well as the precursor flow ratio and total gas amount. They also examined the effect of microwave plasma power and radio-frequency (RF) bias voltage. They found that the key process is to deposit at higher pressures in a microwave/RF PECVD reactor, which provides the simultaneous decrease of n and S_eff,UL_. The latter was found to depend mostly on the defect density of the interface between silicon and SiNx. Furthermore, by optimizing the deposition parameters, a comparatively constant and low S_eff,UL_ was obtained on p and n-type c-Si substrates, with low resistivity in the wide range of n = 1.85–4.07 at 632 nm wavelength.

### 2.5. Post-Deposition Treatment

SiNx layers grown by different types of the CVD method tend to be rich in hydrogen, therefore post-deposition thermal treatment is often applied to reduce the hydrogen content of the films. Heat treatment after layer deposition can take place under different conditions (e.g., with or without vacuum break, in situ/ex situ, and various annealing types, atmospheres, and lengths). Alpuim et al. [46] applied in situ annealing for SiNx thin films with 50 nm thickness prepared by HWCVD on c-Si substrates and investigated the effect of the power density, duration, and pressure of 13.56 MHz Ar plasma treatment on the features of the layer stacks. They found that 2.1 nm as a deposited surface roughness could be decreased to 0.7 nm by applying optimized plasma conditions (30 eV plasma energy, 8 min treatment duration). In contrast, treatment with high energy (100 eV) plasma resulted in 2.7 nm surface roughness, which could be attributed to the damaged surface due to the sputtering of atoms.

An alternative annealing method is the rapid thermal annealing (RTA) method, which involves rapid heating usually provided by an indirect infrared lamp as a heating source for high temperatures (~500–1000 °C) of the deposited layer and substrate. Once the required temperature is reached, it is held for a certain time, typically for 30–60 s. Ren et al. [71] investigated the density of the charge-trapping centers in PECVD SiNx layers as a function of the film stoichiometry and temperature of the post-deposition RTA with a fixed duration of 30 s. They found that as a result of the heat treatment at temperatures varying between 500 and 800 °C, the defect density of the layers increased regardless of the N/Si ratio; however, the most expressed raise was experienced for the sample, which was the most rich in Si. They found that high temperature annealed N-rich layers were more suitable for solar cells as effective surface passivation layers. In terms of the impact of RTA on the optical behavior of SiNx layers, Keita et al. [72] performed a comparative analysis wherein they studied the influence of the annealing atmosphere and temperature on the optical parameters of PECVD SiNx thin films. They considered three different (850, 950, and 1050 °C) RTA temperatures and found that the dielectric function (DF) follows minor variation below 950 °C; however, above this temperature, more stressed change was observed. Comparing the optical properties of the Si nano inclusions of films after RTA treatment revealed that the imaginary DF was increased with the annealing temperature, which could be attributed to the enhanced ability to form crystal lattices at elevated temperatures higher than 950 °C, resulting in more active contributions of Si nanoinclusions to the absorption. Additionally, higher RTA temperature leads to a significant decrease of the gap energies, which should be attributed to structural modifications of the embedded silicon. Finally, the effect of the annealing atmosphere was investigated. It was found that the introduction of oxygen to the argon slightly affected the properties of the films as well as the volume of the Si nanoinclusions; however, the effect of the annealing gas mixture or the precursor gas flow ratio during deposition was much more pronounced.

Jafari et al. [73] performed hydrogen effusion measurements for SiNx:H thin films prepared by PE-CVD from NH_3_ and SiH_4_ gases. Additional FTIR measurements revealed that the peak corresponding to the hydrogen effusion was shifted from 550 °C to 800 °C due to the change of the hydrogen bonding from Si–H to N–H bonds. Furthermore, it was found that the N-gradient SiNx stacking layer showed s 50% less hydrogen evolution rate, which was attributed to the not fully effused hydrogen. Finally, they observed that the annealing process caused a surface change (blistering), which appeared in the form of dark spots with a diameter of roughly 80 µmon as depicted in light microscopy images. In terms of the background of such a layer surface change, the peak related to early hydrogen effusion and the formation of the surface blistering correlated clearly.

## 3. Physical Vapor Deposition

**Figure 2:**
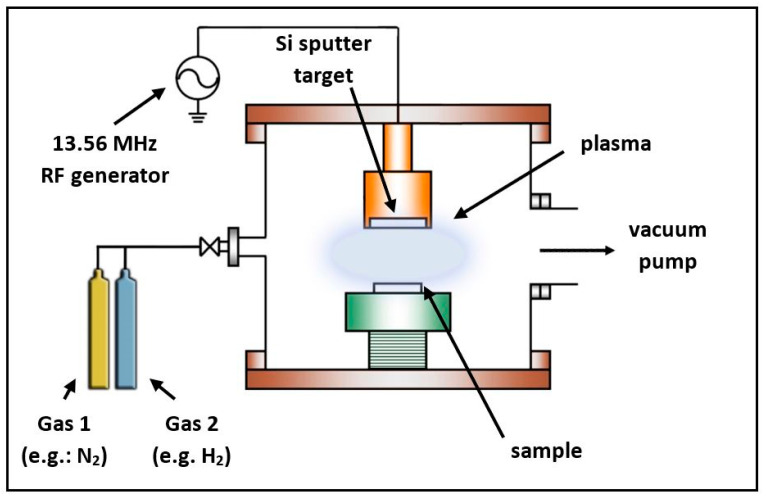
Schematic drawing of an RF sputtering chamber.

Physical vapor deposition (PVD) refers to a method used for thin film deposition in microtechnology and nanotechnology. A common feature of PVD methods is that an initially typically solid or liquid material is transformed to the vapor phase, which then returns to the solid state on the surface of the substrate. PVD can be carried out in several ways. Figure 2 shows the schematic drawing of an RF sputtering chamber.

**Figure 3:**
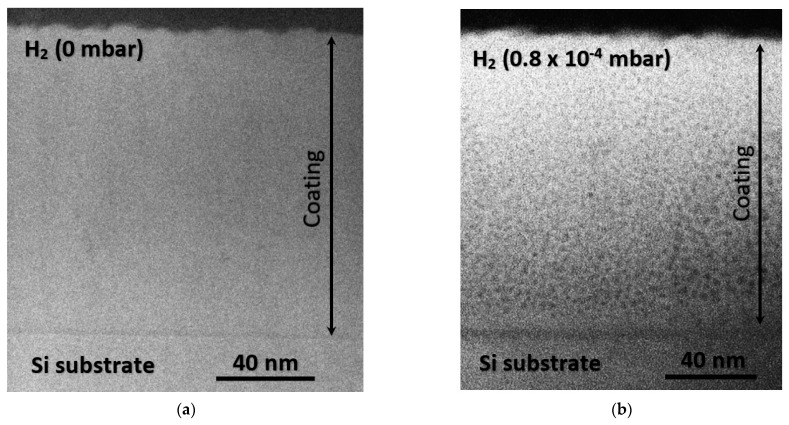
HAADF-STEM images of a-SiN_x_ layers: (**a**) hydrogen-free a-SiN_x_ layer and (**b**) a-SiN_x_:H.

In the case of SiNx deposition, sputtering is the most common technique in which the solid state material (which is called the target in this process) is brought to the vapor phase by means of bombarding the material with electrically charged particles, causing atoms and groups of atoms to escape from its surface. The advantage of sputtering in the SiNx deposition process is the ability to fabricate hydrogen-free layers due to the lack of hydrogen-containing precursor gases. Additionally, this technique also allows for the fabrication of hydrogenated silicon nitride (SiNx:H) films by introducing hydrogen as an additional process gas. It was proven in one of our previous works [74] that the hydrogen incorporation into RF sputtered SiNx layers has a significant effect on the layer porosity. As shown in Figure 3, high-angle annular dark field scanning transmission electron microscopy (HAADF STEM) confirmed denser SiNx films for hydrogen-free sputtering than for the hydrogenated sputtering process, which resulted in a porous structure of the thin films with homogenously distributed nanometer-scale porosities.

### 3.1. Effect of the Power Supply

SiNx layers can be deposited by a sputtering system, utilizing either direct current (DC) or radio frequency (RF) power delivery systems. Dergez et al. characterized SiNx layers sputtered by DC [75] and RF [76] power supplies. They found that in the case of the DC power supply, the deposition rate was proportional to the utilized power. In addition, the power normalized deposition rate was decreased from 0.041 to 0.037, while the back pressure of nitrogen was increased from 3 to 9 µbar during the deposition, which was due to the enhanced number of collisions of the atoms leaving the target. The same behavior of the deposition rate against the power and back pressure of nitrogen was revealed for RF sputtering as well; however, the deposition rate for the RF power supply was found to be lower by a factor of 1.5–2 than the deposition rate for the DC power supply, whose behavior was attributed to the disparate power distribution in the sheath and plasma, known as the “deposition rate paradox” in the literature [77,78,79].

Kiseleva et al. [80] investigated the effect of the power supply characteristics on the properties of sputtered silicon nitride layers. A comparison was performed on thin films obtained by a DC power source and pulsed current power source with 100 and 134 kHz frequencies. They found that the deposition rate decreased while the nitrogen flow increased, regardless of the applied power supply type; however, in the case of low nitrogen flow (~4.5–6 sccm), the pulsed current source with 134 kHz provided a significantly higher deposition rate. The refractive index of the layers decreased from 2.7 to 1.9, while the nitrogen flow was increased from 4.5 to 10.5 sccm for all three types of power sources. Ina addition, clear differences were revealed in the morphology of the thin film surfaces obtained by different power supplies. It was found that in the case of DC power, electrical arcs on the target surface caused droplet fractions formed on the film surface, which may have resulted in the decadence of the efficiency of the film. It was proved that pulsed current mode avoids the formation of droplets.

In certain applications, the simultaneous use of different power supplies and/or target types (co-sputtering) is advantageous to obtain the desired layer performance. So et al. [6] investigated how ultrathin stochiometric SiNxbarrier layers influence the formation and luminescence of Si nanocrystals (NCs) in multilayer stacks, which consist of alternating Si-rich nitride (SRN) and ultrathin Si_3_N_4_ films. They applied the co-sputtering technique from a metal Si target and DC power source, and from a Si_3_N_4_ ceramic target and RF power source to control the Si content (ratio of N to Si atoms) in SRN thin films. Increased DC power of the Si target resulted in higher Si content of the SRN film. Following the deposition of 25 alternating SRN layers with 5 nm thickness and Si_3_N_4_ layers with 1 nm thickness, the multilayer structure was covered by a Si_3_N_4_ layer with 10 nm thickness and was annealed in the N_2_ environment at higher than 900 °C temperatures. It was found that uniformly sized Si NCs were created during the annealing process. Furthermore, the Si_3_N_4_ barrier layers with 1 nm thickness were proved to be able to retain the Si NCs’ formation within the SRN layers. Improved photoluminescence (PL) performance could be related to the enhanced crystallization and nitride passivation in the coatings.

### 3.2. Mechanical Properties

Vila et al. [81] characterized the hardness and Young’s modulus of SiNx layers prepared by reactive sputtering, utilizing pure Si and Si_3_N_4_ sputtering targets as well as Ar/N_2_ gas mixtures. They found that hardness and Young’s modulus were varied in the range of 8–23 and 100–210 GPa, respectively, depending on the preparation parameters. A clear inverse correlation between the oxygen concentration and the mechanical properties was proved, which was attributed to the fact that Si–O bonds in the SiNx layers tended to decrease the hardness and elastic modulus towards the values typical for silicon oxide. Additionally, they investigated a model for estimating the mechanical properties of SiNx from the elastic constants of Si–O and Si–N bonds. The calculations yielded 23.9 and 215 GPa high limits for the hardness and elastic moduli, respectively, which represented oxygen-free pure silicon nitride. Finally, they found that the hardness of the layer can be effectively improved by altering amorphous SiNx into partially crystalline forms, which can be achieved either by applying higher substrate temperatures during the fabrication or by post-deposition annealing. It was revealed that the substrate temperature of 850 °C results in increased hardness and an elastic modulus of up to 23.4 and 220 GPa, which are similar to the values that were foreseen by the abovementioned model for the high limit of mechanical properties. In the case of post-depositon thermal treatment, the vacuum level of the annealing atmosphere could be a limiting factor considering poor vacuum conditions lead to the oxidation of films, thus degrading the mechanical properties. A similar correlation between the nitrogen concentration and the hardness was found by Schmidt et al. [82] who investigated the mechanical properties of SiNx layers deposited by high power impulse magnetron sputtering against the N_2_/Ar gas amount ratio. It was revealed that increasing the N concentration of the films resulted in increased film densities of up to 2.98 g/cm^3^. In parallel, hardness and Young’s modulus also showed an increasing trend, which should be the result of higher SiNx density due to the enhanced N concentration.

### 3.3. Thermal Properties

SiNx thin films with various excess silicon are hopeful candidates for light sources, which are compatible with silicon-based electronics [83]. In such an application device, operation and stability can be enhanced by improving the thermal conductivity, thus reducing the heating of the photonic crystal. Marconnet et al. [84] investigated thermal conductivity by time-domain thermoreflectance (TDTR) measurements of ~400 nm thick amorphous SiNx samples with various excess Si concentrations deposited by nitrogen reactive magnetron sputtering. They found that the thermal conductivity of the samples showed a decreasing trend in the range of 2.66–1.25 W/mK against the silicon concentration. The investigation of the effect of the post-thermal annealing temperature (600–1100 °C) revealed that for a certain level of excess Si concentration (45.5%), the thermal conductivity increased with the increasing annealing temperature.

In magneto-optical (MO) recording applications, amorphous SiNx layers are used as protecting dielectric films for amorphous rare earth-transition metal (RE-TM) coatings, which are the functional layers of the MO medium. In such an application, the process wherein the device writes is a thermal writing process in which thermal cycles are appended to MO disks. As a result of the thermal expansion coefficient difference between the layers and substrates, thermal stress could appear within the thermal cycle. Lai et al. [85] characterized the thermal stress of SiNx films at temperatures varying between 25 and 400 °C. During the heating processes, the stress was almost constant, while after the second thermal cycle, the residual stress switched from compressive (~−780 MPa) to tensile (~1050 MPa).

**Figure 4:**
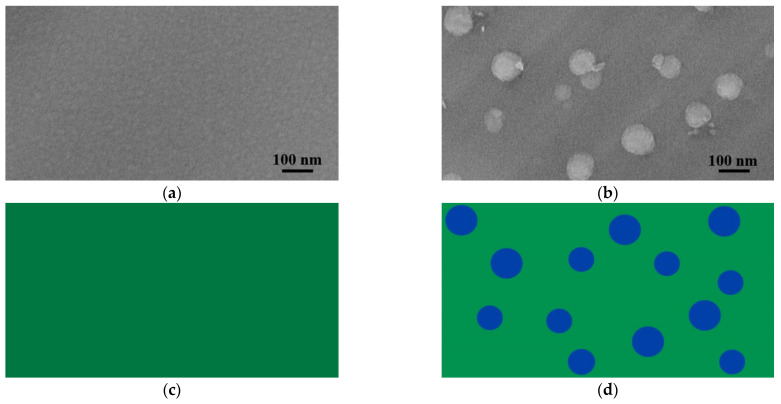
Hydrogen formation in RF-sputtered a-SiNx:H thin films. SEM image of a layer surface (**a**) before annealing and (**b**) after annealing at 65 °C. Schematic representation of the layer surface (**c**) before annealing and (**d**) after annealing.

During heat treatment, a part of the silicon–hydrogen (Si–H) and nitrogen–hydrogen (N–H) bonds of the SiNx:H thin films broke. Consequently, molecular hydrogen was formed, which was then released either to the environment or towards the substrate, playing an important role in the densification of the layer as well as in the formation of its passivation behavior [31]. A similar surface deformation (surface blistering) was found by Jafari et al. [73] for PE-CVD SiNx:H thin films (presented in Section 2.5), wherein RF sputtered SiNx:H thin films at an even lower temperature (~65 °C). Figure 4 presents the scanning electron microscope (SEM) images of the a-SiNx:H layer surfaces prior to and after the heat treatment.

The creation of blisters with a ~100 nm diameter at such a low temperature should be attributed to a similar effect reported by Serényi et al. [86] for a-Si:H layers. Due to the hydrogen and/or nitrogen release from the layer, bubbles filled with gases containing hydrogen and/or nitrogen molecules were created on the layer surface. During annealing, thermal expansion resulted in the increase of the volume of these bubbles, which at a critical point burst caused blister creation on the surface.

### 3.4. Optical Properties

Paule et al. [87] examined the optical behavior of SiNx thin films with thicknesses between 200 and 300 nm, deposited by reactive sputtering, utilizing a pure Si target. They applied N_2_/Ar atmosphere, wherein the total pressure was kept constant at 0.3 Pa, while the P_N2_/(P_N2_ + P_Ar_) relative partial pressure of nitrogen varied between 0 and 0.5. The refractive index at 1000 nm varied between ~3.2 and 2.2, and showed a decreasing trend similar to the absorption coefficient, while the nitrogen flow was increased, which refers to the compositional change of the layers from nitrogen-doped silicon to stochiometric silicon nitride. Strong dependence of the optical behavior of the sputtered SiNx layers on the partial pressure of N_2_ is also supported by the work of Signore et al. [88] who characterized SiNx thin films deposited by RF sputtering from N_2_ and Ar gas mixtures, with the nitrogen flow ratio varying between 10 and 100%. They found that the higher nitrogen content of the gas mixture resulted in the increase of the refractive index at 1800 nm from 1.6 to 1.73, and this behavior was assigned to the presence of (oxygen and hydrogen) contamination. The possibility of tuning the refractive index of the SiNx layer by the modification of the nitrogen flow enables the fabrication of multilayer structures of silicon nitrides with different refractive indices, providing the antireflection effect for solar cell applications.

Although the refractive index of silicon nitride is usually tuned by adjusting the nitrogen and argon gas flow ratio, other deposition parameters could also have significant effects on the refractive index of the layer. Guruvenket et al. [89] studied direct current (DC) magnetron sputtered SiNx layers and explored how the substrate bias voltage influences the refractive index, measured at 650 nm. They found that the refractive index varied between ~2.04 and ~1.87, while the bias voltage was changed from 0 to −120 V, and cathode current density as well as the nitrogen partial pressure were kept constant at the value of 2.5 mA/cm^2^ and 3 × 10^−2^ Pa, respectively. It was revealed that the increase of the bias voltage from 0 to −40 V first caused the decrease of the refractive index from ~2.04; afterwards, it was saturated at a value of about 1.92.

**Figure 5:**
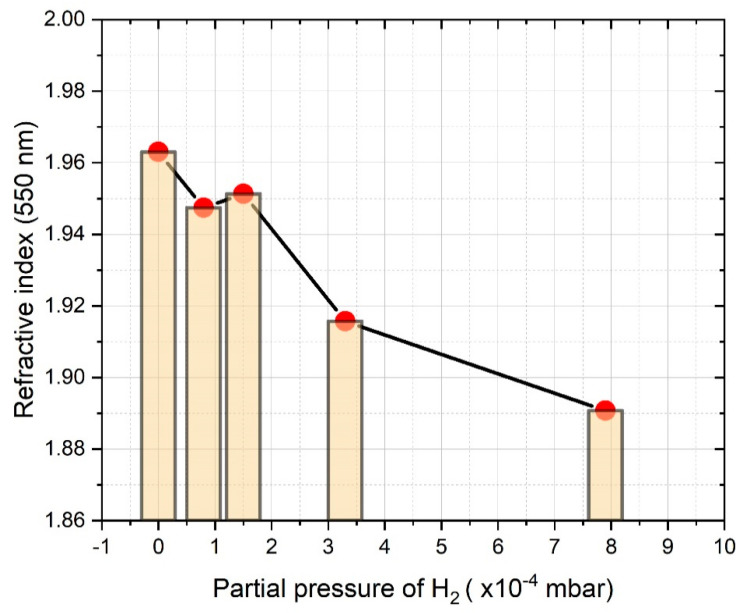
Refractive index at 550 nm against H_2_ partial pressure.

In the case of the deposition of SiNx:H thin films by the introduction of molecular hydrogen to the chamber, control of the hydrogen pressure serves as an alternative technique for tuning the refractive index of the thin films. Mokkedem et al. [90] studied the correlation of the hydrogen gas pressure and the refractive index of DC magnetron sputtered SiNx:H layers. It was revealed that when the partial pressure of H_2_ increased from 4.5 to 9 mPa, the refractive index showed a decreasing trend from 1.92 to 1.78. Considering that, in parallel, the increase of the nitrogen to silicon ([N]/[Si]) ratio from 1.03 to 1.22, as well as the increase of the hydrogen to silicon ([H]/[Si]) ratio from 1.47 to 1.65 were proved, the experienced variations of the refractive index should be attributed to the incorporation of H and N atoms into the layers. We experienced a similar correlation between the partial pressure of the hydrogen applied to the chamber and the refractive index at 550 nm of SiNx:H layers prepared by RF sputtering, as shown in Figure 5 [74].

The refractive index at 550 nm of the layers decreased from 1.96 to 1.89, while the partial pressure of H_2_ was increased from 0 to 7.9 × 10^−4^ mbar. These variations of the refractive index should be attributed to the incorporation of H and N atoms into the layers [90]. Table 2 presents the comparison of the mechanical, thermal, and optical properties of CVD, PVD, and ALD SiNx layers.

## 4. ALD

The atomic layer deposition (ALD) is a subclass of CVD based on sequential gas-phase chemical processes. Since this method allows for low temperature deposition as well as for the control of the film thickness with precision in the atomic scale, it has attracted great scientific interest concerning SiNx layer fabrication in recent years. In this section, current research progress, the most important trends, and future prospects are summarized. 

### 4.1. Thermal ALD

Thermal ALD relies on the heating of the deposition chamber and the substrate to drive the surface reaction kinetics; therefore, higher deposition temperature (typically above 450 °C) is required. In the majority of the related works, chlorosilanes as silicon-containing precursors and ammonia as a nitrogen source are applied [94,95,96,97,98,99]. Additionally, Morishita et al. [100] revealed that SiNx can be also deposited by thermal ALD from Si_2_Cl_6_ between temperatures of 525 and 650 °C. It should be also noted that we are not aware of any thermal ALD SiNx which was produced from non-chlorosilane-based precursors. Riedel et al. [98] investigated thermal ALD SiNx layers deposited at various substrate temperatures varying from 310 to 500 °C, utilizing octachlorotrisilane (OCTS, Si_3_Cl_8_) as an alternative Si-containing precursor gas and NH_3_ as a N source. They found that the wet etch rates in diluted HF (100:1–0.49%) decreased versus the raising substrate temperature and thus increasing film density. Significant amounts of oxygen were also found, the quantity of which shows a decreasing trend with an increasing deposition temperature. In the absence of an oxygen source, we can assume that the layers were oxidizing because of the contact with the ambient air. Park et al. [97] also found that thermal ALD SiNx layers are non-stochiometric and can be simply oxidized by air exposures, leading to approximately 7–8 atomic % O content of the thin film.

### 4.2. PE ALD

**Figure 6:**
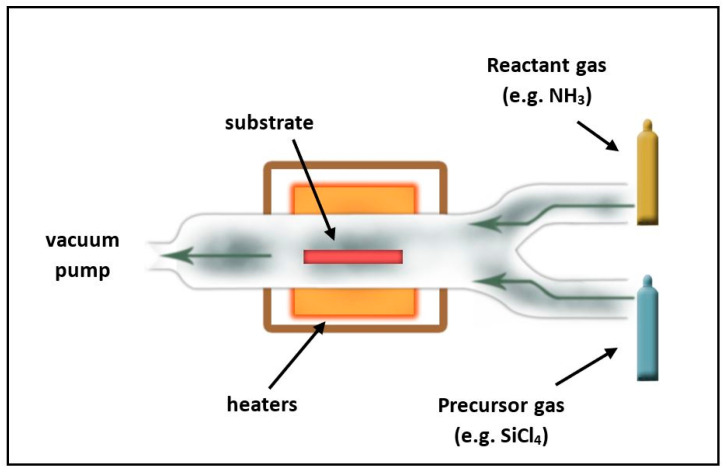
Schematic drawing of a PE-ALD reactor.

In order to overwhelm the difficulties of the increased deposition temperature of thermal ALD methods and to meet the requirements of modern (e.g., interconnect and spacer) applications [93,100,101], plasma-enhanced ALD is utilized in several cases. This technique was proved to be an appropriate method to deposit silicon nitride at T < 400 °C by several researchers [102,103,104,105]. Furthermore, the enhanced reactivity of the plasma allows for the application of precursors which don’t contain halogen atoms [106,107,108,109]. Deposition temperature can be further decreased by remotely generated plasma, which is then transported to the chamber [110,111,112,113,114,115]. In Figure 6, a schematic drawing of a PE-ALD reactor is presented.

In addition to the lower deposition temperature, this method is also advantageous in minimizing plasma-induced damages and surface nucleation time, as well as in avoiding undesirable gas-phase reactions. Several reports studied how the substrate temperature influences the PE ALD process. Andringa et al. [116] characterized the refractive index and the chemical composition of SiNx moisture permeation barrier layers fabricated by PE ALD with the SiH_2_(NH^t^BU)_2_ precursor and by nitrogen-fed plasma at different deposition temperatures. It was revealed that the refractive index (at 633 nm) was increased from 1.8 to 1.9, while the deposition temperature was raised from 80 to 200 °C. In contrast, carbon, oxygen, and hydrogen impurity levels showed decreasing trends against increasing deposition temperatures. In terms of porosity, they found that there were no open pores with diameters bigger than 0.3 nm independently from the deposition temperature. Jang et al. [108] studied the effect of temperature on SiNx deposition by PE-ALD from trisilylamine [TSA, (SiH_3_)_3_N] and NH_3_ in the range of 250–350 °C. However, it was revealed that all films are near-stochiometric and the N/Si stochiometric ratio slightly increases (from 1.32 to 1.35) due to the increase of the deposition temperature (from 250 to 350 °C). Higher temperatures resulted in higher refractive indices as well as lower hydrogen contents. The effect of the temperature on the defect density was also proved since increased deposition temperatures led to enhanced trap densities, which should be attributed to the lower hydrogen content. The latter behavior allows for adjusting the defect densities to meet the requirements of charge trap flash memory applications by controlling the fabrication temperature. Another work [117] introduces low-temperature (250–300 °C) PE-ALD of SiNx, utilizing neopentasilane [NPS, (SiH_3_)_4_Si] with a direct N_2_ plasma. The thin film deposition was compared to a more frequently used source gas, specifically trisilylamine [TSA, (SiH_3_)_3_N], as a reference. In terms of the growth behavior and N_2_ plasma saturation, no significant differences were found. However, higher growth rates were observed for NPS. It was revealed that increased N_2_ plasma exposure time caused a decrease in the refractive index for both precursors. Koehler et al. [118] investigated SiNx thin films deposited at higher temperatures (400–500 °C) by ALD for spacer and gate encapsulation applications. They found that the SiNx film quality and growth conditions have important roles in shaping the performance of high-k metal gate technology.

## 5. Conclusions

A combination of advantageous layer properties establishes SiNx thin films a promising candidate for several application fields. In this work, an overview of the latest published works for SiNx thin films was presented with a focus on the applications and obtainable layer properties by applying different deposition methods. The latest achievements of CVD and PVD depositions technologies were highlighted with a comparison of their characteristic mechanical, thermal, and optical properties. In view of the growing demand for ultrathin SiNx layers with precise control of the composition, a group of CVD methods (ALCVD) was reviewed in a separate section.

In terms of deposition temperature, a trend towards lower processing temperatures was observed due to efforts to minimize the damage of thermally instable substrates, such as IC applications or polymer materials of OLED devices. Another trend of the development of SiNx thin films concerned targeting to achieve better mechanical properties, driven by hard coating applications under challenging environmental conditions.

Obviously, SiNx layer properties are affected by several parameters such as the fabrication method, precursor gas chemistry, type of power supply used for the plasma generation, and the substrate temperature. However, the exact correlation between the process parameters and the layer properties could depend on the actual deposition equipment. The results reviewed in this paper could act as a guideline for the development and further tuning of SiNx layer properties to meet the expectations of certain applications.

## Figures and Tables

**Table 1:** Thermal conductivity of widely used materials in microelectronics devices.

Material	Thermal Conductivity (Wm^−1^K^−1^)
SiNx	3.2 [53]
a-Si	1.7–2.24 [54]
a-SiO_2_	1.1–1.26 [55]
a-HfO_2_	0.49–0.95 [56]
a-Al_2_O_3_	1.18–1.70 [57]
a-TiO_2_	0.7–1.7 [58]

**Table 2:** Comparison of mechanical, thermal, and optical properties of CVD and PVD SiNx thin films.

Layer Properties	CVD	PVD	ALD
**Mechanical** **properties**	-	-	-
Deposition stress	135–235 MPa [48]	-	700–1300 GPa [91]
Hardness	28.0 ± 2.3 GPa [48] 16.1–19.8 GPa [49] 13–23 GPa [50]	8–23 GPa [81]	-
Young’s modulus	256 GPa [52]	100–210 GPa [81]	-
Poisson’s ratio	0.28 [52]	-	-
**Thermal** **properties**	-	-	-
Thermal conductivity	$3.2\pm 0.5\;{\mathrm{Wm}}^{-1}K^{-1}$ [53] $2.0–2.5{\;Wm}^{-1}K^{-1}$ [60] ~0.25–~0.7 ${\mathrm{Wm}}^{-1}K^{-1}$ [61] 0.8–1.7 ${\mathrm{Wm}}^{-1}K^{-1}$ [62]	2.66–1.25 ${\mathrm{Wm}}^{-1}K^{-1}$ [84]	-
Thermal diffusivity	$1.3\times 10^{-2}\pm 1\times 10^{-3}\;{\mathrm{cm}}^{2}s^{-1}$ [53]	-	-
Heat capacity	$0.7\pm 0.1{\;\mathrm{Jg}}^{-1}K^{-1}$ [53]	-	-
**Optical** **properties**	-	-	-
Refractive index	1.96–1.8 [64] ~2.7 to–1.6 [65] ~1.91–~1.98 [66] 2.05–2.11 @632.8 nm [67] 1.95 to 3.35 @633 nm [68]	3.2–2.2 @1000 nm [87] 1.6–1.73 @1800 nm [88] 2.04–1.87 @650 nm [89] 1.92–1.78 [90] 1.96–1.89 @550 nm [74]	1.86–2.0 @633 nm [91] ~1.77–1.87 @633 nm [92] 2.01 [93]

## Data Availability

The data presented in this study are available on request from the corresponding author.

## References

[1] Kopfer J.M., Keipert-Colberg S., Borchert D. (2011). Capacitance–voltage characterization of silicon oxide and silicon nitride coatings as passivation layers for crystalline silicon solar cells and investigation of their stability against x-radiation. Thin Solid Films.

[2] Li D., Kunz T., Wolf N., Liebig J.P., Wittmann S., Ahmad T., Hessmann M.T., Auer R., Göken M., Brabec C.J. (2015). Silicon nitride and intrinsic amorphous silicon double antireflection coatings for thin-film solar cells on foreign substrates. Thin Solid Films.

[3] Hernandez J., Allebe C., Tous L., John J., Poortmans J. Laser ablation and contact formation for Cu-plated large area C-silicon industrial solar cells. Proceedings of the 35th IEEE Photovoltaic Specialists Conference.

[4] Bailly M.S., Karas J., Jain H., Dauksher W.J., Bowden S. (2016). Damage-free laser patterning of silicon nitride on textured crystalline silicon using an amorphous silicon etch mask for Ni/Cu plated silicon solar cells. Thin Solid Films.

[5] Iwahashi T., Morishima M., Fujibayashi T., Yang R., Lin J., Matsunaga D. (2015). Silicon nitride anti-reflection coating on the glass and transparent conductive oxide interface for thin film solar cells and modules. J. Appl. Phys..

[6] So Y.H., Huang S., Conibeer G., Green M.A. (2011). Formation and photoluminescence of Si nanocrystals in controlled multilayer structure comprising of Si-rich nitride and ultrathin silicon nitride barrier layers. Thin Solid Films.

[7] Green B., Chu K., Chumbes E., Smart J., Shealy J., Eastman L. (2000). The effect of surface passivation on the microwave characteristics of undoped AlGaN/GaN HEMTs. IEEE Electron Device Lett..

[8] Fagerlind M., Allerstam F., Sveinbjörnsson E.O., Rorsman N., Kakanakova-Georgieva A., Lundskog A., Forsberg U., Janzen E. (2010). Investigation of the interface between silicon nitride passivations and AlGaN/AlN/GaN heterostructures by C(V) characterization of metal-insulator-semiconductor-heterostructure capacitors. J. Appl. Phys..

[9] Chovan J., Uherek F. (2018). Photonic Integrated Circuits for Communication Systems. Radioengineering.

[10] Sharma T., Wang J., Kaushik B.K., Cheng Z., Kumar R., Zhao W., Li X. (2020). Review of Recent Progress on Silicon Nitride-based Photonic Integrated Circuits. IEEE Access.

[11] Frigg A., Boes A., Ren G., Abdo I., Choi D.Y., Gees S., Mitchell A. (2019). Low loss CMOS-compatible silicon nitride photonics utilizing reactive sputtered thin films. Opt. Express.

[12] Mine T., Fujisaki K., Ishida T., Shimamoto Y., Yamada R., Torii K. (2007). Electron Trap Characteristics of Silicon Rich Silicon Nitride Thin Films. Jpn. J. Appl. Phys..

[13] Tiron V., Velicu I.-L., Pana I., Cristea D., Rusu B.G., Dinca P., Porosnicu C., Grigore E., Munteanu D., Tascu S. (2018). HiPIMS deposition of silicon nitride for solar cell application. Surf. Coatings Technol..

[14] Pettersson M., Tkachenko S., Schmidt S., Berlind T., Jacobson S., Hultman L., Engqvist H., Persson C. (2013). Mechanical and tribological behavior of silicon nitride and silicon carbon nitride coatings for total joint replacements. J. Mech. Behav. Biomed. Mater..

[15] Kulczyk-Malecka J., Kelly P., West G., Clarke G.C.B., Ridealgh J. (2013). Diffusion studies in magnetron sputter deposited silicon nitride films. Surf. Coatings Technol..

[16] Dressler W., Riedel R. (1997). Progress in silicon based non-oxide structural ceramics. Int. J. Refract. Metals Hard Mater..

[17] Jiang J.Z., Kragh F., Frost D., Lindelov H. (2001). Hardness and thermal stability of cubic silicon nitride. J. Physics Condens. Matter.

[18] Ku S.-L., Lee C.-C. (2010). Optical and structural properties of silicon nitride thin films prepared by ion-assisted deposition. Opt. Mater..

[19] Budaguan B.G., Stryahilev D.A., Aivazov A.A. (1997). Optical properties, statistics of bond angle deformations and density of states in Si-rich a-SiNx: H alloys. J. Non-Cryst. Solids.

[20] Vargheese K.D., Rao G.M. (2001). Electrical properties of silicon nitride films prepared by electron cyclotron resonance assisted sputter deposition. J. Vac. Sci. Technol. A.

[21] Verlaan V., van der Werf C.H.M., Houweling Z.S., Romijn I.G., Weeber A.W., Dekkers H.F.W., Goldbach H.D., Schropp R.E.I. (2007). Multi-crystalline Si solar cells with very fast deposited (180 nm/min) passivating hot-wire CVD silicon nitride as antireflection coating. Prog. Photovoltaics Res. Appl..

[22] Deshpande S.V., Gulari E., Brown S.W., Rand S.C. (1995). Optical properties of silicon nitride films deposited by hot filament chemical vapor deposition. J. Appl. Phys..

[23] Kessels W.M.M., Hong J., van Assche F.J.H., MOschner J.D., Lauinger T., Soppe W.J., Weeber A.W., Schram D.C., van de Sanden M.C.M. (2002). High-rate deposition of a-SiNx:H for photovoltaic application y the expanding thermal plasma. J. Vac. Sci. Technol. A.

[24] Martınez F.L., Ruiz-Merino R., Del Prado A., San Andrés E., Mártil I., González-Dıaz G., Jeynes C., Barradas N.P., Wang L., Reehal H.S. (2004). Bonding structure and hydrogen content in silicon nitride thin films deposited by electron cyclotron resonance plasma method. Thin Solid Films.

[25] San Andrés E., Del Prado A., Martınez F.L., Mártil I., Bravo D., López F.J. (2000). Rapid thermal annealing effects on the structural properties and density of defects in SiO_2_ and SiNx:H films deposited by electron cyclotron resonance. J. Appl. Phys..

[26] Martil I., del Prado A., San Andres E., Gonzalez Daz G., Martnez F.L. (2003). Rapid thermally annealed plasma deposited SiNx:H thin films: Application to metal-insulator-semiconductor structures with Si, In0.53 Ga0.47 As, and InP. J. Appl. Phys..

[27] Martnez F.L., del Prado A., Martil I., Gonzalez-Daz G., Selle B., Sieber I. (1999). Thermally induced changes in the optical properties of SiNx:H films deposited by the electron cyclotron resonance plasma method. J. Appl. Phys..

[28] Bommali R.K., Ghosh S., Khan S.A., Srivastava P. (2018). Hydrogen loss and its improved retention in hydrogen plasma treated a-SiNx:H films:ERDA study with 100 MeV ag7+ ions. Nucl. Instrum. Methods Phys. Res. B.

[29] Yoo J., So J., Yu G., Yi J. (2011). Study on hydrogenated silicon nitride for application of high efficiency crystalline silicon solar cells. Sol. Energy Mater. Sol. Cells.

[30] Fitzner M., Abelson J., Kanicki J. (2011). Investigation of Hydrogen and Nitrogen Thermal Stability in PECVD a-Sinx:H. MRS Online Proc. Libr. Arch..

[31] Santos-Filho P., Stevens G., Lu Z., Koh K., Lucovsky G. (1995). Hydrogen Release and Si-N Bond-Healing Infrared Study of Rapid Thermal Annealed Amorphous Silicon Nitride Thin Films. MRS Online Proc. Libr..

[32] Edmonds M., Sardashti K., Wolf S., Chagarov E., Clemons M., Kent T., Park J.H., Tang K., McIntyre P.C., Yoshida N. (2017). Low temperature thermal ALD of a SiNx interfacial diffusion barrier and interface passivation layer on SixGe1−x(001) and SixGe1− x(110). J. Chem. Phys..

[33] Nakajima A., Khosru Q.D.M., Yoshimoto T., Yokoyama S. (2002). Atomic-layer-deposited silicon-nitride/SiO2 stack—A highly potential gate dielectrics for advanced CMOS technology. Microelectron. Reliab..

[34] Zhu S., Nakajima A. (2007). Atomic Layer Deposition of HfO2and Si Nitride on Ge Substrates. Jpn. J. Appl. Phys..

[35] Cho H., Lee N., Choi H., Park H., Jung C., Song S., Yuk H., Kim Y., Kim J.-W., Kim K. (2019). Remote Plasma Atomic Layer Deposition of SiNx Using Cyclosilazane and H2/N2 Plasma. Appl. Sci..

[36] Murray C.A., Elliott S.D., Hausmann D., Henri J., Lavoie A. (2014). Effect of Reaction Mechanism on Precursor Exposure Time in Atomic Layer Deposition of Silicon Oxide and Silicon Nitride. ACS Appl. Mater. Interfaces.

[37] Eom T.-K., Kim S.-H., Kang D.-H., Kim H. (2011). Characteristics of Plasma-Enhanced Atomic Layer Deposited RuSiN as a Diffusion Barrier against Cu. J. Electrochem. Soc..

[38] Eom T.K., Kim S.H., Park K.S., Kim S., Kim H. (2011). Formation of Nano-Crystalline Ru-Based Ternary Thin Films by Plasma-Enhanced Atomic Layer Deposition. Electrochem. Solid-State Lett..

[39] Kaloyeros A.E., Jove F.A., Goff J., Arkles B. (2017). Review—Silicon Nitride and Silicon Nitride-Rich Thin Film Technologies: Trends in Deposition Techniques and Related Applications. ECS J. Solid State Sci. Technol..

[40] Kaloyeros A.E., Pan Y., Goff J., Arkles B. (2020). Review—Silicon Nitride and Silicon Nitride-Rich Thin Film Technologies: State-of-the-Art Processing Technologies, Properties, and Applications. ECS J. Solid State Sci. Technol..

[41] Torchynska T.V., Espinola J.C., Hernandez E.V., Khomenkova L., Delachat F., Slaoui A. (2015). Effect of the stoichiometry of Si-rich silicon nitride thin films on their photoluminescence and structural properties. Thin Solid Films.

[42] Lee K.D., Dahiwale S.S., Do Kim Y., Kim S., Bae S., Park S., Tark S.J., Kim D. (2012). Influence of gas mixture ratio on properties of SiNx:H films for Crystalline Silicon Solar Cells. Energy Procedia.

[43] Wenas W.W., Winata T., Barmawi M. Growth study of wide bandgap a-Si:H and a-SiN:H by PECVD method for application in thin film transistor. Proceedings of the ICSE 2000, 2000 IEEE International Conference on Semiconductor Electronics, Proceedings (Cat. No.00EX425).

[44] Vet B., Zeman M. (2010). Comparison of a SiC:H and a-SiN:H as candidate materials for a p-i interface layer in a-Si:H p-i-n solar cells. Energy Procedia.

[45] Jhansirani K., Dubey R., More M., Singh S. (2016). Deposition of silicon nitride films using chemical vapor deposition for photovoltaic applications. Results Phys..

[46] Alpuim P., Majee S., Cerqueira M., Tondelier D., Geffroy B., Bonnassieux Y., Bourée J. (2015). Effect of argon ion energy on the performance of silicon nitride multilayer permeation barriers grown by hot-wire CVD on polymers. Thin Solid Films.

[47] French P.J., Sarro P.M., Mallée R., Fakkeldij E.J.M., Wolffenbuttel R.F. (1997). Optimization of a low-stress silicon nitride process for surface-micromachining applications. Sens. Actuators.

[48] Toivola Y., Thurn J., Cook R., Cibuzar G., Roberts K. (2003). Influence of deposition conditions on mechanical properties of low-pressure chemical vapor deposited low-stress silicon nitride films. J. Appl. Phys..

[49] Taylor J.A. (1991). The mechanical properties and microstructure of plasma enhanced chemical vapor deposited silicon nitride thin films. J. Vac. Sci. Technol. A.

[50] King S., Chu R., Xu G., Huening J. (2010). Intrinsic stress effect on fracture toughness of plasma enhanced chemical vapor de-posited SiNx:H films. Thin Solid Films.

[51] Link A., Sooryakumar R., Bandhu R.S., Antonelli G.A. (2006). Brillouin light scattering studies of the mechanical properties of ultrathin low-k dielectric films. J. Appl. Phys..

[52] Carlotti G., Colpani P., Piccolo D., Santucci S., Senez V., Socino G., Verdini L. (2002). Measurement of the elastic and viscoelas-tic properties of dielectric films used in microelectronics. Thin Solid Films.

[53] Mastrangelo C.H., Tai Y., Muller R.S. (1990). Thermophysical Properties of Low-residual Stress, Silicon-rich, PLCVD Silicon Nitride Films. Sens. Actuators A Phys..

[54] Regner K.T., Sellan D.P., Su Z., Amon C.H., McGaughey A.J., Malen J. (2013). Broadband phonon mean free path contributions to thermal conductivity measured using frequency domain thermoreflectance. Nat. Commun..

[55] Käding O.W., Skurk H., Goodson K.E. (1994). Thermal conduction in metallized silicon-dioxide layers on silicon. Appl. Phys. Lett..

[56] Panzer M.A., Shandalov M., Rowlette J.A., Oshima Y., Chen Y.W., McIntyre C., Goodson K.E. (2009). Thermal Proper-ties of Ultrathin Hafnium Oxide Gate Dielectric Films. IEEE Electron Device Letters.

[57] Gorham C.S., Gaskins J.T., Parsons G.N., Losego M.D., Hopkins P.E. (2014). Density dependence of the room tempera-ture thermal conductivity of atomic layer deposition-grown amorphous alumina (Al2O3). Appl. Phys. Lett..

[58] Mun J., Kim S.W., Kato R., Hatta I., Lee S.H., Kang K.H. (2007). Measurement of the thermal conductivity of TiO2 thin films by using the thermo-reflectance method. Thermochim. Acta.

[59] Griffin A.J., Brotzen F.R., Loos P.J. (1994). Effect of thickness on the transverse thermal conductivity of thin dielectric films. J. Appl. Phys..

[60] Griffin A.J., Brotzen F.R., Loos P.J. (1994). The effective transverse thermal conductivity of amorphous Si3N4 thin films. J. Appl. Phys..

[61] Lee S.-M., Cahill D.G. (1997). Heat transport in thin dielectric films. J. Appl. Phys..

[62] Bogner M., Hofer A., Benstetter G., Gruber H., Fu R.Y. (2015). Differential 3ω method for measuring thermal conductivity of AlN and Si3N4 thin films. Thin Solid Films.

[63] Lube T., Dusza J. (2007). A silicon nitride reference material—A testing program of ESIS TC6. J. Eur. Ceram. Soc..

[64] Joshi B.C., Eranna G., Runthala D.P., Dixit B.B., Wadhawan O.P., Vyas P.D. (2000). LPCVD and PECVD silicon nitride for microelectronics technology. Indian J. Eng. Mater. Sci..

[65] Lowe A.J., Powell M.J., Elliott S.R. (1986). The elctronic properties of plasmadeposited films of hydrogenated amorphous SiNx (0 < x < 1.2). J. Appl. Phys..

[66] Maeda M., Arita Y. (1982). Electrical properties and their thermal stability for silicon nitride films prepared by plasmaenhanced deposition. J. Appl. Phys..

[67] Mei J.J., Chen H., Shen W.Z., Dekkers H.F.W. (2006). Optical properties and local bonding configurations of hydrogenated amorphous silicon nitride thin films. J. Appl. Phys..

[68] Charifi H., Slaoui A., Stoquert J.P., Chaib H., Hannour A. (2016). Opto-Structural Properties of Silicon Nitride Thin Films De-posited by ECR-PECVD. World J. Condens. Matter Phys..

[69] Lelièvre J.F., Fourmond E., Kaminski A., Palais O., Ballutaud D., Lemiti M. (2009). Study of the composition of hydrogenated silicon nitride SiNx:H for efficient surface and bulk passivation of silicon. Sol. Energy Mater. Sol. Cells.

[70] Wan Y., McIntosh K.R., Thomson A.F. (2013). Characterisation and optimisation of PECVD SiNx as an antireflection coating and passivation layer for silicon solar cells. AIP Adv..

[71] Ren Y., Weber K.J., Nursam N.M., Wang D. (2010). Effect of deposition conditions and thermal annealing on the charge trap-ping properties of SiNx films. Appl. Phys. Lett..

[72] Keita A.-S., Naciri A.E., Delachat F., Carrada M., Ferblantier G., Slaoui A., Stchakovsky M. (2010). Dielectric functions of PECVD-grown silicon nanoscale inclusions within rapid thermal annealed silicon-rich silicon nitride films. Thin Solid Films.

[73] Jafari S., Hirsch J., Lausch D., John M., Bernhard N., Meyer S. (2019). Composition limited hydrogen effusion rate of a-SiNx:H passivation stack. AIP Conf. Proc..

[74] Hegedüs N., Lovics R., Serényi M., Zolnai Z., Petrik P., Mihály J., Fogarassy Z., Balázsi C., Balázsi K. (2021). Examination of the Hydrogen Incorporation into Radio Frequency-Sputtered Hydrogenated SiN_x_ Thin Films. Coatings.

[75] Dergez D., Schneider M., Bittner A., Schmid U. (2015). Mechanical and Electrical Properties of DC Magnetron Sputter Depos-ited Amorphous Silicon Nitride Thin Films. Thin Solid Films.

[76] Dergez D., Schneider M., Bittner A., Pawlak N., Schmid U. (2016). Mechanical and electrical properties of RF magnetron sput-ter deposited amorphous silicon-rich silicon nitride thin films. Thin Solid Films.

[77] Nyaiesh A., Holland L. (1981). The dependence of deposition rate on power input for dc and rf magnetron sputtering. Vacuum.

[78] Este G., Westwood W.D. (1988). A quasi-direct-current sputtering technique for the deposition of dielectrics at enhanced rates. J. Vac. Sci. Technol. A.

[79] Maniv S. (1991). A comparison of deposition rates and temperature measurements for dc and rf diode sputtering. J. Appl. Phys..

[80] Kiseleva D.V., Yurjev Y., Petrakov Y.V., Sidelev D., Korzhenko D., Erofeev E.V. (2017). Study on the influence of the magnetron power supply on the properties of the Silicon Nitride films. J. Phys. Conf. Ser..

[81] Vila M., Cáceres D., Prieto C. (2003). Mechanical properties of sputtered silicon nitride thin films. J. Appl. Phys..

[82] Schmidt S., Hänninen T., Goyenola C., Wissting J., Jensen J., Hultman L., Goebbels N., Tobler M., Högberg H. (2016). SiN Coatings Deposited by Reactive High Power Impulse Magnetron Sputtering: Process Parameters influencing the Nitrogen Content. Appl. Mater. Interfaces.

[83] Yerci S., Li R., Kucheyev S.O., van Buuren T.O.N.Y., Basu S.N., Dal Negro L. (2009). Energy transfer and 1.54 um emission in amor-phous silicon nitride films. Appl. Phys. Lett..

[84] Marconnet A., Panzer M., Yerci S., Minissale S., Wang X., Zhang X., Negro L.D., Goodson K.E. (2012). Thermal conductivity and photoluminescence of light-emitting silicon nitride films. Appl. Phys. Lett..

[85] Lai C.H., Huang C.L., Hsu C.Y., Lin I.N., Jou J. (2012). Optical and thermal properties of SiNx for MO disks. Proceedings of SPIE—The International Society for Optical Engineering.

[86] Serényi M., Csík A., Hámori A., Kalas B., Lukács I., Zolnai Z.S., Frigeri C. (2019). Diffusion and reaction kinetics governing surface blistering in radio frequency sputtered hydrogenated a-SixGe1-x (0 ≤ x ≤ 1) thin films. Thin Solid Films.

[87] Paule E., Elizalde E., Martínez-Duart J.M., Albella J.M. (1987). Optical properties of reactively sputtered silicon nitride thin films. Vacuum.

[88] Signore M., Sytchkova A., Dimaio D., Cappello A., Rizzo A. (2011). Deposition of silicon nitride thin films by RF magnetron sputtering: A material and growth process study. Opt. Mater..

[89] Guruvenket S., Ghatak J., Satyam P., Rao G.M. (2005). Characterization of bias magnetron-sputtered silicon nitride films. Thin Solid Films.

[90] Mokeddem K., Aoucher M., Smail T. (2006). Hydrogenated amorphous silicon nitride deposited by DC magnetron sputtering. Superlattices Microstruct..

[91] Jhang P.-C., Lu C.-P., Shieh J.-Y., Yang L.-W., Yang T., Chen K.-C., Lu C.-Y. (2017). Properties of N-rich Silicon Nitride Film Deposited by Plasma-Enhanced Atomic Layer Deposition. Solid-State Electron..

[92] Meng X., Kim H.S., Lucero A.T., Hwang S.M., Lee J.S., Byun Y.-C., Kim J., Hwang B.K., Zhou X., Young J. (2018). Hollow Cathode Plasma-Enhanced Atomic Layer Deposition of Silicon Nitride Using Pentachlorodisilane. ACS Appl. Mater. Interfaces.

[93] Klaus J., Ott A., Dillon A., George S. (1998). Atomic layer controlled growth of Si3N4 films using sequential surface reactions. Surf. Sci..

[94] Yokoyama S., Ikeda N., Kajikawa K., Nakashima Y. (1998). Atomic-layer selective deposition of silicon nitride on hydro-gen-terminated Si surfaces. Appl. Surf. Sci..

[95] Hansch W., Nakajima A., Yokoyama S. (1999). Characterization of silicon/oxide/nitride layers by x-ray photoelectron spec-troscopy. Appl. Phys. Lett..

[96] Nakajima A., Khosru Q.D.M., Yoshimoto T., Kidera T., Yokoyama S. (2002). Low-temperature formation of highly reliable silicon-nitride gate dielectrics with suppressed soft-breakdown phenomena for advanced complementary met-al-oxide-semiconductor technology. J. Vac. Sci. Technol..

[97] Park K., Yun W.D., Choi B.J., Kim H.D., Lee W.J., Rha S.K., Park C.O. (2009). Growth studies and characterization of sili-con nitride thin films deposited by alternating exposures to Si2Cl6 and NH3. Thin Solid Films.

[98] Riedel S., Sundqvist J., Gumprecht T. (2015). Low temperature deposition of silicon nitride using Si3Cl8. Thin Solid Films.

[99] Yusup L.L., Park J.-M., Noh Y.-H., Kim S.-J., Lee W.-J., Park S., Kwon Y.-K. (2016). Reactivity of different surface sites with silicon chlorides during atomic layer deposition of silicon nitride. RSC Adv..

[100] Morishita S., Sugahara S., Matsumura M. (1997). Atomic-layer chemical-vapor-deposition of silicon-nitride. Appl. Surf. Sci..

[101] Nakajima A., Khosru Q.D.M., Yoshimoto T., Kidera T., Yokoyama S. (2002). NH_3_-annealed atomic-layer-deposited silicon nitride as a high-k gate dielectric with high reliability. Appl. Phys. Lett..

[102] Goto H., Shibahara K., Yokoyama S. (1996). Atomic layer controlled deposition of silicon nitride with self-limiting mechanism. Appl. Phys. Lett..

[103] Yokoyama S., Goto H., Miyamoto T., Ikeda N., Shibahara K. (1997). Atomic layer controlled deposition of silicon nitride and in situ growth observation by infrared reflection absorption spectroscopy. Appl. Surf. Sci..

[104] Knoops H.C., Braeken E.M., de Peuter K., Potts S.E., Haukka S., Pore V., Kessels W.M. (2015). Atomic Layer Deposi-tion of Silicon Nitride from Bis(tertbutylamino)silane and N2 plasma. Appl. Mater. Interfaces.

[105] Ovanesyan R.A., Hausmann D.M., Agarwal S. (2015). Low-Temperature Conformal Atomic Layer Deposition of SiNx Films Using Si2Cl6 and NH3 Plasma. ACS Appl. Mater. Interfaces.

[106] King S.W. (2011). Plasma enhanced atomic layer deposition of SiNx:H and SiO2. J. Vac. Sci. Technol. A.

[107] Triyoso D.H., Hempel K., Ohsiek S., Jaschke V., Shu J., Mutas S., Dittmar K., Schaeffer J., Utess D., Lenski M. (2013). Evaluation of Low Temperature Silicon Nitride Spacer for High-k Metal Gate Integration. ECS J. Solid State Sci. Technol..

[108] Jang W., Jeon H., Kang C., Song H., Park J., Kim H., Seo H., Leskela M., Jeon H. (2014). Temperature dependence of silicon ni-tride deposited by remote plasma atomic layer deposition. Phys. Status Solidi A.

[109] Knoops H.C.M., De Peuter K.K., Kessels W.E. (2015). Redeposition in plasma-assisted atomic layer deposition: Silicon nitride film quality ruled by the gas residence time. Appl. Phys. Lett..

[110] van Assche F.J., Unnikrishnan S., Michels J.J., van Mol A.M., van de Weijer P., van de Sanden M.C., Creatore M. (2014). On the intrinsic moisture permeation rate of remote microwave plasma-deposited silicon nitride layers. Thin Solid Films.

[111] Kim Y., Provine J., Walch S.P., Park J., Phuthong W., Dadlani A.L., Kim H.-J., Schindler P., Kim K., Prinz F.B. (2016). Plasma Enhanced Atomic Layer Deposition of SiN-AlN Composites for Ultra Low Wet Etch Rates in Hydrofluoric Acid. Appl. Mater. Interfaces.

[112] Provine J., Schindler P., Kim Y., Walch S.P., Kim H., Kim K.-H., Prinz F.B. (2016). Correlation of film density and wet etch rate in hydrofluoric acid of plasma enhanced atomic layer deposited silicon nitride. AIP Adv..

[113] Park J.-M., Jang S.J., Yusup L.L., Lee W.-J., Lee S.-I. (2016). Plasma-Enhanced Atomic Layer Deposition of Silicon Nitride Using a Novel Silylamine Precursor. ACS Appl. Mater. Interfaces.

[114] Suh S., Ryu S.W., Cho S., Kim J.R., Kim S., Hwang C.S., Kim H.J. (2016). Low-temperature SiON films deposited by plas-ma-enhanced atomic layer deposition method using activated silicon precursor. J. Vac. Sci. Technol. A.

[115] Ande C.K., Knoops H.C.M., de Peuter K., van Drunen M., Elliott S.D., Kessels W.M.M. (2015). Role of Surface Termination in Atomic Layer Deposition of Silicon Nitride. J. Phys. Chem. Lett..

[116] Andringa A.M., Perrotta A., de Peuter K., Knoops H.C., Kessels W.M., Creatore M. (2015). Low-Temperature Plas-ma-Assisted Atomic Layer Deposition of Silicon Nitride Moisture Permeation Barrier Layers. Appl. Mater. Interfaces.

[117] Weeks S., Nowling G., Fuchigami N., Bowes M., Littau K. (2016). Plasma enhanced atomic layer deposition of silicon nitride using neopentasilane. J. Vac. Sci. Technol. A.

[118] Koehler F., Triyoso D.H., Hussain I., Antonioli B., Hempel K. (2014). Challanges in spacer process development for leading edge high-k metal gate technology. Phys. Status Solidi C.

